# Rab6 regulates recycling and retrograde trafficking of MR1 molecules

**DOI:** 10.1038/s41598-020-77563-4

**Published:** 2020-11-27

**Authors:** Megan E. Huber, Regina Kurapova, Chelsea M. Heisler, Elham Karamooz, Fikadu G. Tafesse, Melanie J. Harriff

**Affiliations:** 1 Department of Pulmonary and Critical Care Medicine, Oregon Health & Science University, Portland, OR 97239 USA; 2 VA Portland Health Care System, 3710 SW US Veterans Hospital Rd, R&D11, Portland, OR 97239 USA; 3 Department of Molecular Microbiology and Immunology, Oregon Health & Science University, Portland, OR 97239 USA

**Keywords:** Antigen processing and presentation, MHC class I

## Abstract

Mucosal-associated invariant T (MAIT) cells are an innate-like T cell subset important in the early response to bacterial and viral lung pathogens. MAIT cells recognize bacterial small molecule metabolites presented on the Class I-like molecule MR1. As with other Class I and Class II molecules, MR1 can likely sample ligands in the intracellular environment through multiple cellular pathways. Rab6, a small GTPase that regulates a number of endosomal trafficking pathways including retrograde transport to the trans-Golgi network (TGN), is involved in the presentation of ligands from *Mycobacterium tuberculosis* (Mtb) to MAIT cells. The Rab6-mediated trafficking pathway contains endosomal compartments that share features with the Mtb intracellular compartment. Using inducible expression of MR1, this study demonstrates that Rab6 regulates the recycling of MR1 molecules from the cell surface through endosomal trafficking compartments to the TGN. This Rab6-dependent pool of recycled MR1, which is available for reloading with ligands from bacterial pathogens like Mtb, may be important for early recognition of infected cells by MAIT cells in the lung.

## Introduction

According to the 2016 World Health Organization report, lower respiratory infections are the 4th leading cause of mortality worldwide^1^. CD8^+^ T cells, which recognize and kill infected target cells, are particularly important to containment of infection with intracellular bacterial lung pathogens such as *Mycobacterium tuberculosis* (Mtb)^2,3^, which by itself is the 10th leading cause of worldwide mortality^1^. Mucosal associated invariant T (MAIT) cells are a subset of cytotoxic T cells that recognize small molecule metabolites produced from vitamin B biosynthetic pathways present in numerous bacteria and fungi^4–6^. Animal models demonstrate a role for MAIT cells in the early response to bacterial lung pathogens such as *Francisella tularensis*^7,8^, *Klebsiella pneumoniae*^9^, and *Legionella longbeachae*^10^. This function is likely due to the relative enrichment of MAIT cells in the mucosal tissues^4,6,11,12^ and their ability to exhibit immediate effector function^13^ in response to bacterially-infected cells.

MAIT cells recognize bacterial small molecule ligands presented on the non-classical MHC Class I-like molecule MR1^5,14^. MR1 is ubiquitously expressed in MHC Class II-positive and -negative mammalian cells, including airway epithelial cells^15,16^. Although studies on the immune response to Mtb typically focus on infected macrophages and dendritic cells, airway epithelial cells are susceptible to Mtb infection and are very efficient at presenting ligands to MAIT cells^17^. The expression of MR1 in these cells and the proximity of MAIT cells to the airway epithelium suggests they could act as sentinels following exposure to Mtb or other pulmonary pathogens. Taken together, the presumed abundance of MAIT cell ligands, the ubiquitous expression of MR1 in mammalian cells, and the enrichment of MAIT cells in mucosal tissues suggest a requirement for tight regulation of MR1 loading and presentation to prevent inappropriate activation of inflammatory immune responses. However, the mechanisms regulating presentation of bacterial MAIT cell ligands on MR1 are not fully understood.

Due to the nature of the small molecule MAIT cell ligands, loading of MR1 does not require classical antigen processing pathways such as those utilized for loading of peptide ligands on classical MHC Class I molecules^4,18,19^. Also distinct from other Class I molecules, MR1 has a primarily intracellular localization in the endoplasmic reticulum (ER) and endosomal compartments^18,20–22^ and translocates to the cell surface upon addition of ligand^21,22^. While there is evidence that MR1 surface expression requires the MHC Class II chaperone, invariant chain^18^, this does not explain MR1 antigen presentation in cell types like airway epithelial cells that do not express MHC Class II or invariant chain. The current model using the ligands 6-formylpterin (6-FP), acetyl-6-formypterin (Ac-6-FP), and 5-OP-RU demonstrates that MR1 is loaded in the ER in C1R cells (reviewed in^23^). However, there are additional pathways governing the loading of bacterially derived ligands that are distinct from loading of exogenously added synthetic ligands and make use of endosomal MR1^21,24^. In a subsequent report, we identified 6-FP-loaded MR1 as permissive for ligand exchange, providing a stabilized pool of MR1 for loading of exogenous ligands in airway epithelial cells^25^. We previously showed that endosome-mediated pathways are utilized in the presentation of ligands from Mtb on MR1^21^, suggesting a role of endocytosis and recycling of MR1 in the presentation of ligands from intracellular microbes. This current study highlights the role of the small GTPase Rab6 in distinct pathways of MR1-dependent antigen presentation. We generated a bronchial epithelial cell line with inducible expression of MR1 to explore the trafficking of newly synthesized or preexisting MR1 proteins. This control of MR1 synthesis enabled deeper understanding of the differential pathways for ER-residing or endosomal compartment-residing MR1, respectively, to translocate to the cell surface for antigen presentation. We demonstrate here that Rab6 regulates the recycling and retrograde trafficking of MR1 through endosomal pathways, but does not impact the translocation of MR1 to the cell surface. Our data is also consistent with a model where MR1 residing in non-ER compartments is available for loading with new ligand. The intracellular localization of Mtb in the endosomal trafficking pathway suggests Mtb ligands can access MR1 through this Rab6-mediated pathway.

## Results

### MR1 expression kinetics using an inducible promoter

**Figure 1:**
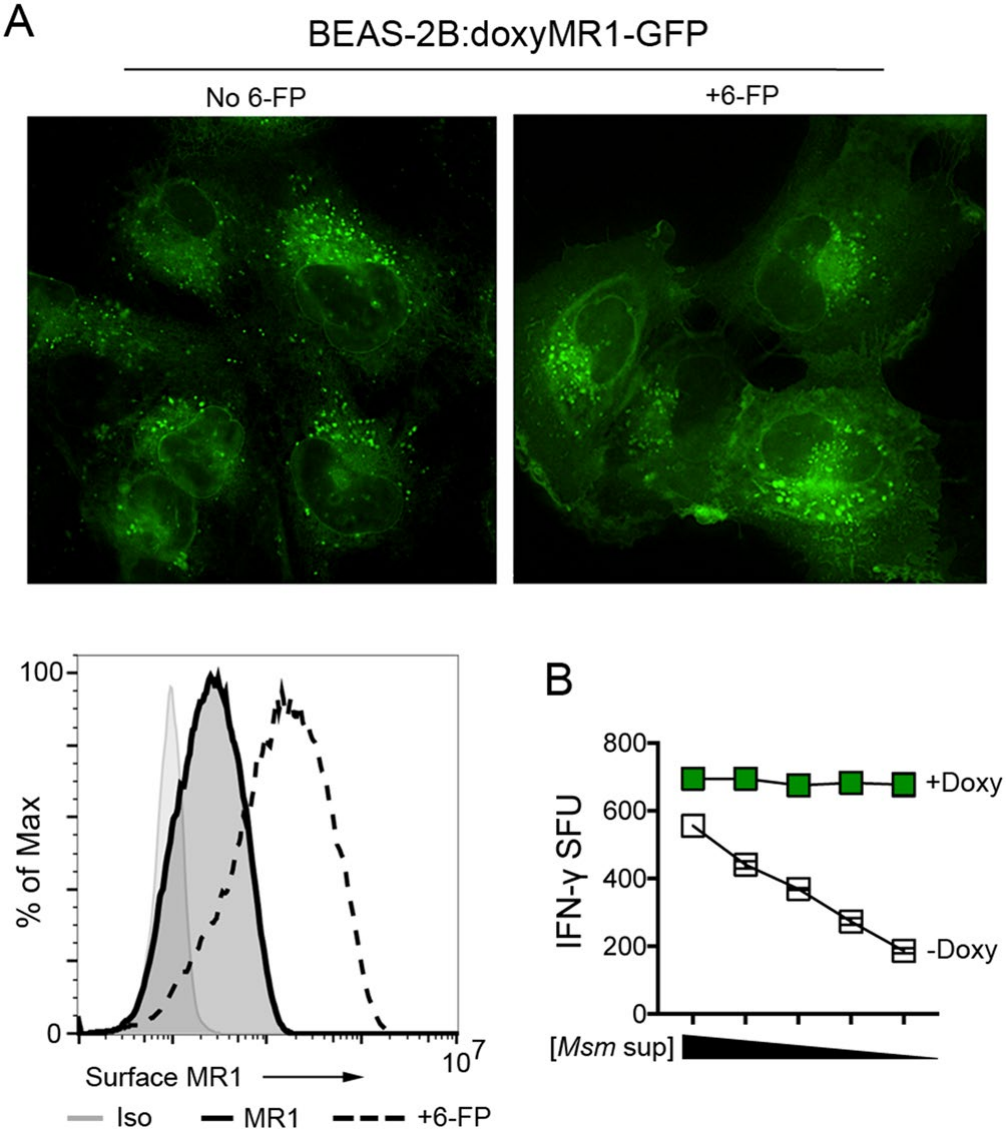
MR1 expressed behind an inducible promoter in stably transduced BEAS-2B cells is functional. (**A**) BEAS-2B:doxyMR1-GFP cells were treated with doxy for 24 h, then incubated for 16 h with 100uM 6-FP. Top: Fluorescence microscopy was used to visualize the localization of MR1 in live cells. Bottom: Flow cytometry was used to visualize the stabilization of MR1 on the cell surface using the 26.5 anti-MR1 antibody (MR1) after treatment with 6-FP (+ 6-FP) or isotype control (Iso). (**B**) BEAS-2B:doxyMR1-GFP cells treated with doxy or media control were incubated in an ELISPOT assay with 0.625-10ul of *M. smegmatis* supernatant and the MAIT cell clone D426G11. MAIT cell response was measured by IFN-gamma spot forming units (SFU). Results shown are representative of at least three independent experiments.

Silencing Rab6 in the bronchial epithelial cell line BEAS-2B resulted in reduced MAIT cell responses to Mtb infection^21^. We previously demonstrated an increase in the number of MR1^+^ endosomal vesicles in Rab6-silenced cells; however, the translocation of MR1 to the cell surface with 6-FP treatment was not affected^21^. This study used cells constitutively over-expressing MR1, which made it difficult to ascertain how Rab6 silencing specifically impacted MR1 expression and function in different cellular compartments. Additionally, although MAIT cell responses in ELISPOT assays clearly demonstrate that MR1 molecules loaded with Mtb ligands reach the cell surface in BEAS-2B cells, there are no measurable changes in the cellular localization and surface translocation of MR1 in the context of Mtb infection using flow cytometry or fluorescence microscopy, consistent with the findings of others^22^. In contrast to the mechanisms that have been identified for loading of exogenously added ligands such as 6-FP and 5-OP-RU^22^, this has created a challenge in identifying mechanisms for the loading of bacterial ligands generated during intracellular infection. To address these concerns, we generated a cell line with inducible MR1 expression. Using a previously described construct^25^, BEAS-2B cells were stably transduced to express MR1 fused to GFP behind a doxycycline (doxy)-inducible promoter. Consistent with transient transfection and induction of the doxyMR1-GFP construct^25^, addition of doxy to the culture media resulted in MR1-GFP expression in the ER and endosomal compartments and translocation of MR1 to the cell surface upon addition of 6-FP (Fig. 1A). Additionally, MAIT cell response to doxy-treated BEAS-2B:doxyMR1-GFP cells stimulated with *Mycobacterium smegmatis* supernatant was increased compared to BEAS-2B:doxyMR1-GFP cells not treated with doxy (expressing wild-type levels of MR1) (Fig. 1B), which confirms that the doxy-inducible MR1-GFP construct is capable of antigen presentation and activation of MAIT cells. Together these results suggest the MR1 in BEAS-2B:doxMR1-GFP cells traffics and functions similarly to endogenous and constitutively over-expressed MR1.

**Figure 2:**
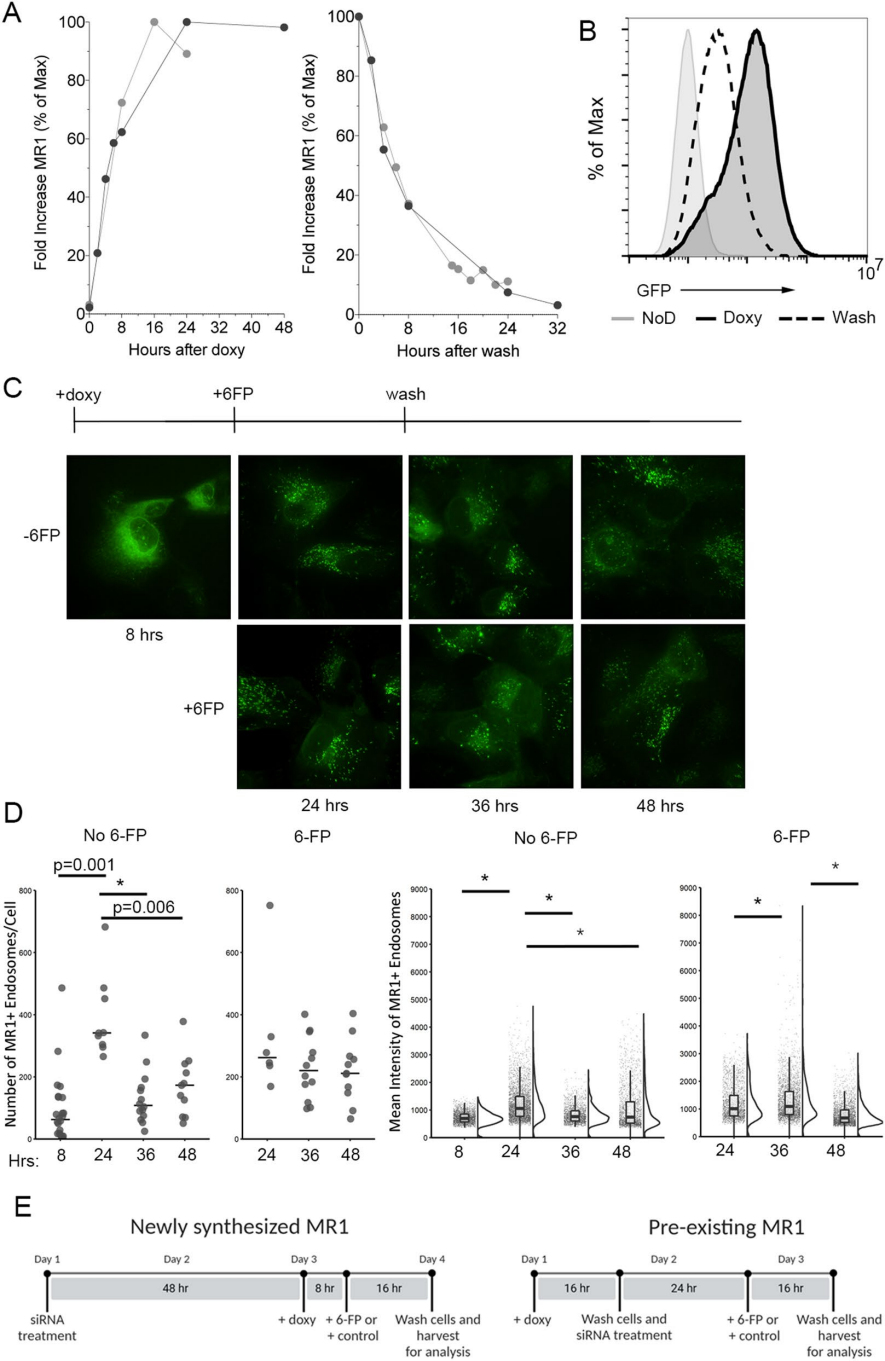
MR1 expression kinetics using an inducible promoter. (**A**) BEAS-2B:doxyMR1-GFP cells were treated with doxy for the indicated times before RNA extraction and RT-PCR. The left panel indicates the fold-increase in *MR1* transcripts over the no doxy control, when the doxy is not removed from the well. The right panel indicates the decline in *MR1* transcripts in the 24 h following the removal and washing of doxy from the wells. Each line is a representative independent experiment. (**B**) MR1-GFP protein expression was measured by flow cytometry in BEAS-2B:doxyMR1-GFP cells treated with or without doxy for 24 h before washing and incubation for an additional day. (**C**) MR1-GFP protein expression was observed by fluorescence microscopy following the addition of doxy for 24hrs, 6FP for 16 h, then subsequent removal and washing of the doxy from the wells for an additional 12 and 24 h. (**D**) Images from (**C**) were analyzed with Imaris to quantify (left) the number of MR1^+^ endosomal compartments per cell and (right) the mean fluorescence intensity of each MR1^+^ compartment. For B-D results shown are representative of at least three independent experiments. * Indicates p value < 0.001. (**E**) Schematic demonstrating the timing of MR1-GFP induction, washing of doxy, addition of 6-FP, and Rab6 siRNA transfection.

RT-PCR analysis of *MR1* gene expression following addition and subsequent removal of the doxy was performed to determine the kinetics of MR1 overexpression in these cells. Increased *MR1* expression peaked at 16–24 h following doxy addition, and remained at these levels for at least an additional 24 h (Fig. 2A, left). Removing doxy by washing cells and replacing the media resulted in a decrease in *MR1* gene expression, returning to near pre-doxy levels by 16–24 h post wash (Fig. 2A, right). Analysis of these cells by flow cytometry and fluorescence microscopy revealed the kinetics of MR1-GFP protein expression. Flow cytometry demonstrated a substantial decrease in total cellular MR1-GFP protein expression 24 h after washing doxy from the media (Fig. 2B), mirroring the RT-PCR results.

BEAS-2B:doxyMR1-GFP cells treated with doxy were imaged in parallel to the RNA expression experiments in Fig. 2A to determine the intracellular localization of MR1 (Fig. 2C, top) and in some cases were treated with 6-FP to determine the surface translocation of MR1 (Fig. 2C, bottom). Images were analyzed using Imaris to quantify GFP endosomal compartments. Early after doxy addition (8 h), MR1 localized predominantly in the ER. By 24 h following doxy treatment, MR1 was observed in post-ER endosomal compartments (p = 0.001) and remained in the ER to a lesser extent (Fig. 2C,D, top, 24 h). Similar to the results from Fig. 2B, MR1-GFP signal was dimmer after washing doxy from the cells for 12 and 24 h (36 and 48 h, top, p < 0.001) and there were fewer MR1^+^ endosomal compartments per cell (36 and 48 h, p < 0.001 and p = 0.006). In cells treated with 6-FP, MR1 was observed on the cell surface at all three timepoints with little change in the overall number of MR1^+^ endosomal compartments per cell (Fig. 2C bottom, D). Previous evidence suggests that 6-FP protects MR1 from degradation^25^, which may explain the increase in MR1 fluorescence in 6-FP-treated cells 12 h after washing doxy (Fig. 2C,D, 36 h, p < 0.001). In contrast, the number and brightness of MR1^+^ endosomal compartments peaks at 24 h after addition of doxy in cells not treated with 6-FP. There are multiple possibilities for this pattern, but the observation confirmed that in our cells, MR1 translocates from the ER to endosome-like cellular compartments following initial synthesis without the addition of exogenous ligand. After 6-FP-driven surface translocation and subsequent removal of ligand, MR1 is capable of recycling into multiple cellular regions including the ER and endosomal compartments.

Taken together, these data validate that the BEAS-2B:doxyMR1-GFP cells can be used to analyze the impact of Rab6 on MR1 surface translocation in the context of “newly synthesized” versus “preexisting” MR1 (Fig. 2E). Specifically, by manipulating Rab6 expression or function prior to the addition of doxy to the media, we can analyze the impact of Rab6 on newly synthesized, predominantly ER-localized MR1 (Fig. 2E, left). In addition, by manipulating Rab6 expression or function at specific time points after the removal of doxy from the media, we could analyze the impact of Rab6 on preexisting MR1 residing in endosomal compartments and on the cell surface (Fig. 2E, right). The temporal control afforded by the BEAS-2B:doxyMR1-GFP cells is key to exploring if Rab6 functions in the translocation of antigen-bound MR1 from the ER to the cell surface (newly synthesized timing) or in the cycling of antigen-bound MR1 between endosomal compartments and the cell surface (preexisting timing).

### Rab6 regulates total and surface expression of preexisting MR1

**Figure 3:**
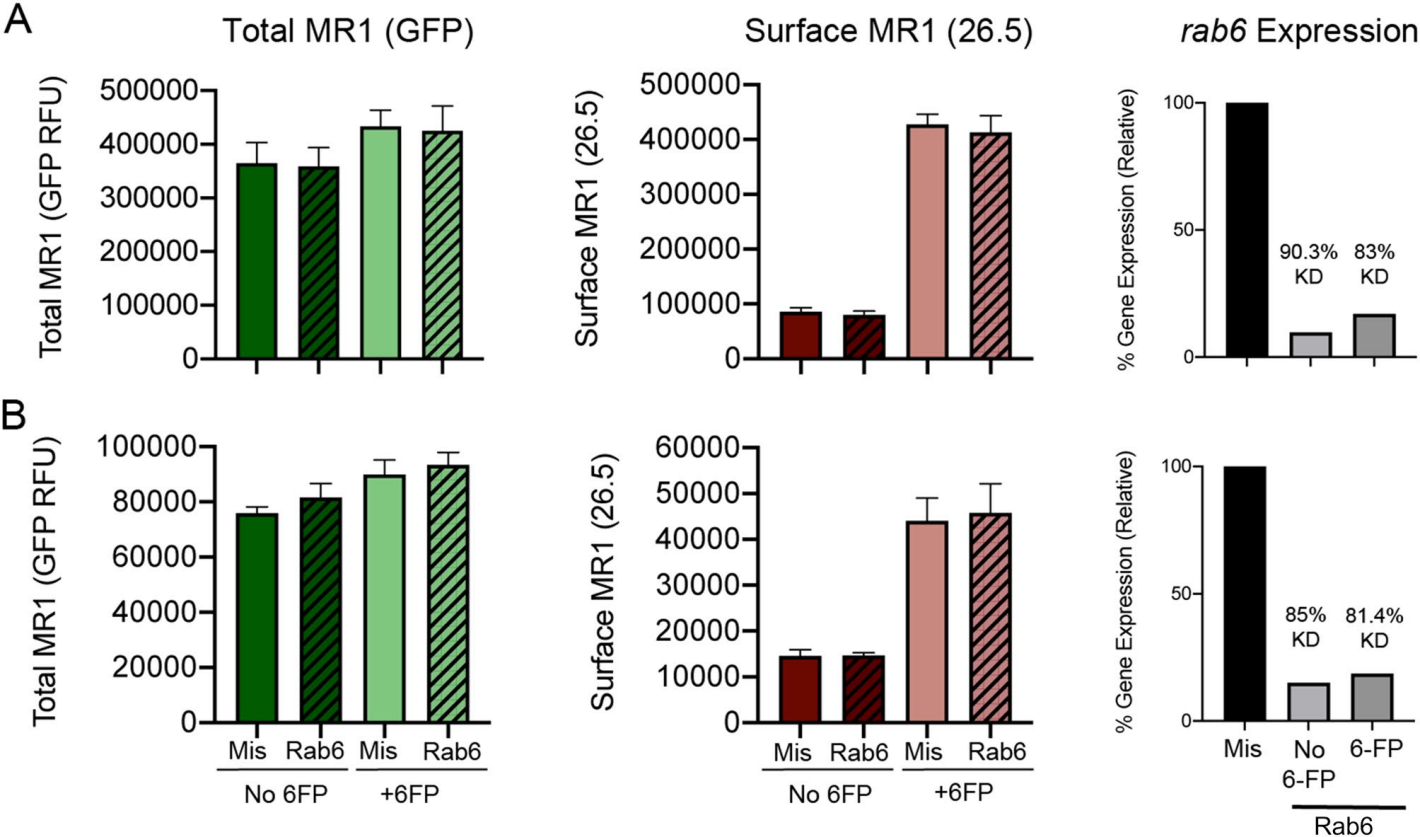
Rab6 silencing impacts the expression and surface translocation of preexisting MR1. (**A**) BEAS-2B:doxyMR1-GFP cells were treated with Rab6 or missense control (Mis) siRNA, doxy, and 6-FP where indicated as per the “Newly synthesized MR1” scheme in Fig. 2e. Live cells were stained for surface MR1, then immediately analyzed by flow cytometry for total (GFP) and surface (26.5) MR1. Rab6 silencing efficiency was validated by RT-PCR (*rab6* Expression). (**B**) BEAS-2B:doxyMR1-GFP cells were treated with Rab6 or missense control (Mis) siRNA, doxy, and 6-FP where indicated as per the “Preexisting MR1” scheme in Fig. 2e. Cells were stained and analyzed as described in A. Results are the geometric mean and standard deviation of the relative fluorescence for each condition normalized to the missense condition from 4 independent experiments.

We examined the expression of total cellular or surface MR1 in Rab6-silenced BEAS-2B:doxyMR1-GFP cells by flow cytometry using the timings from Fig. 2E. In cells where Rab6 was silenced prior to inducing MR1 expression (i.e. “newly synthesized” MR1) with no addition of exogenous ligand, there were no differences in total MR1 as measured by GFP signal, or surface MR1 as measured by surface staining with the α-MR1 26.5 antibody (Fig. 3A). In cells expressing preexisting MR1, there was an increase in total MR1 in Rab6-silenced cells at steady state (Fig. 3B). Although the increase was not statistically significant, it was repeatable and not observed in cells expressing newly synthesized MR1. Additionally, it was consistent with our previous results using a system with constitutive overexpression of MR1^21^. There was no corresponding surface MR1 increase in Rab6-silenced cells expressing preexisting MR1 (Fig. 3A,B). Since Rab6 silencing did not impact expression of or translocation of newly synthesized MR1 to the cell surface, we hypothesized that the observed changes resulting from Rab6 silencing impacted preexisting MR1.

We next examined whether Rab6 silencing affected total and surface MR1 expression in the context of the exogenously added ligand, 6-FP. First, we silenced Rab6 before doxy induction of MR1 and incubation with 6-FP to look at the impact of silencing Rab6 on newly synthesized MR1. Similar to our previous observations in Fig. 2C,D and in^25^, cells that were incubated with 6-FP showed an increase in total MR1 which suggests that 6-FP stabilizes MR1 molecules (Fig. 3A). Addition of 6-FP to the culture equally increased total expression and surface translocation of MR1 in Rab6-silenced cells compared to the missense control (Fig. 3A). These data demonstrate that Rab6 does not regulate loading or egress of newly synthesized MR1 from the ER in the context of 6-FP. Next, we silenced Rab6 in cells containing preexisting MR1, then added 6-FP. Similar to the steady state preexisting MR1 condition, there was a small but consistent increase in total MR1 and no increase in surface stabilized MR1 in Rab6-silenced cells compared to missense control when cells were incubated with 6-FP (Fig. 3B). Taken together, these data suggest that Rab6 could play a role in MR1 trafficking, but not at the level of translocation to the cell surface. Furthermore, these data provide supporting evidence that extracellular ligands like 6-FP can be loaded onto MR1 that has not been newly synthesized^25^.

### Rab6 regulates MR1 recycling from the cell surface

**Figure 4:**
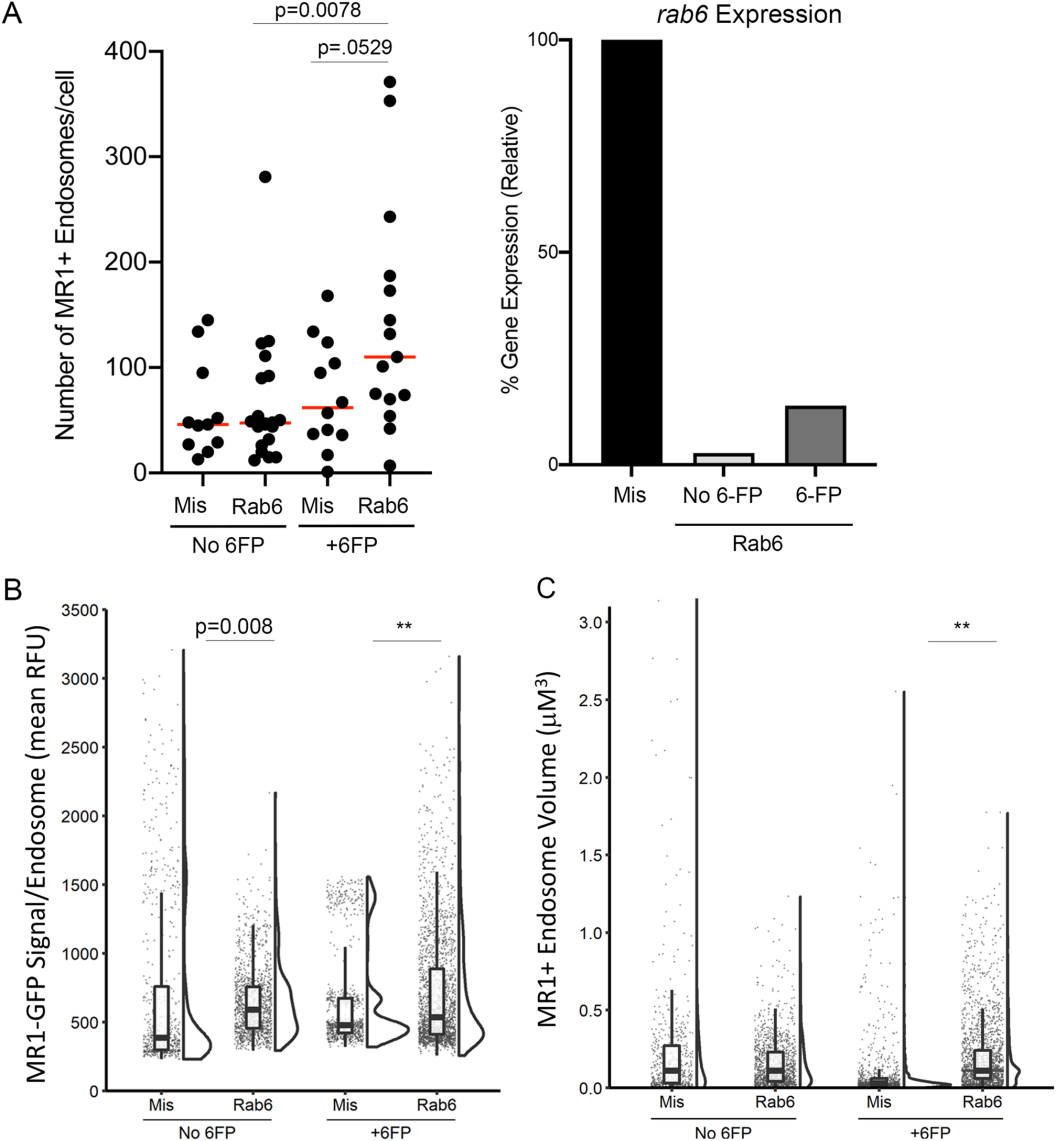
Rab6 silencing impacts the localization of MR1 to endosomal compartments. BEAS-2B:doxyMR1-GFP cells were treated by newly synthesized (**A**) or preexisting (**B**) MR1 experimental timings as in Fig. 2e. Rab6 silencing efficiency was validated by RT-PCR. Cells were imaged live and the number of MR1^+^ endosomal compartments, the mean relative fluorescence of the MR1 in those compartments, and the volume of the compartments were quantified using Imaris. Each dot represents an individual cell or MR1^+^ endosomal compartment. *Indicates p value < 0.001. Results are representative of at least three independent experiments.

Because we observed small but statistically insignificant changes to total cellular MR1 or surface MR1 by flow cytometry, we next used fluorescence microscopy to observe whether there were any changes to the intracellular localization of MR1 in Rab6-silenced cells. In cells expressing newly synthesized or preexisting MR1-GFP, we quantified the number of endosome-like compartments containing MR1 and the relative amount of MR1 in these structures. As we previously showed in cells constitutively overexpressing MR1-GFP^21^, we observed an increase in the number of MR1^+^ endosomes in Rab6-silenced cells expressing newly synthesized MR1 (Fig. 4A). Interestingly, there was significantly less MR1-GFP in each endosome (p = 0.003) and the endosomes were smaller (p < 0.001) in this condition. In the context of 6-FP, the same differences between control and Rab6-silenced cells was observed, but to a lesser extent (Fig. 4B). This is more apparent in cells incubated without 6-FP, since translocation from the cell surface was not impacted by the presence of an exogenous antigen. Together, these results support the hypothesis that Rab6 impacts endosomal MR1 trafficking rather than surface translocation of MR1 from the ER.

In cells expressing preexisting MR1, the number of MR1^+^ endosomal compartments per cell in control and Rab6-silenced cells was not significantly different at steady state or after incubation with 6-FP (Fig. 4B). Therefore, as above, we examined the relative amount of MR1-GFP fluorescence signal in these compartments. At both steady state and in the context of 6FP treatment, we observed a different distribution in the MR1-GFP signal among endosomal compartments (Fig. 4B), as evidenced by the multimodal distribution of MR1-GFP signal in control compared to Rab6-silenced cells. Analysis of the distribution of the data showed there was a higher mean MR1-GFP signal in endosomal compartments in Rab6-silenced cells (p < 0.001) and the distribution of the MR1-GFP signal was distinct between conditions. In support of this, there was also a significant decrease in the mean volume of MR1^+^ endosomal compartments in Rab6-silenced cells, with or without the addition of 6-FP (Fig. 4C, p < 0.001). This again suggested an impairment in recycling of endocytosed MR1 or in endocytosis of MR1 following surface stabilization in Rab6-silenced cells, such that MR1 follows different endosomal trafficking pathways resulting in distinct intracellular distribution.

### Rab6 regulates transport of MR1 molecules to the TGN

**Figure 5:**
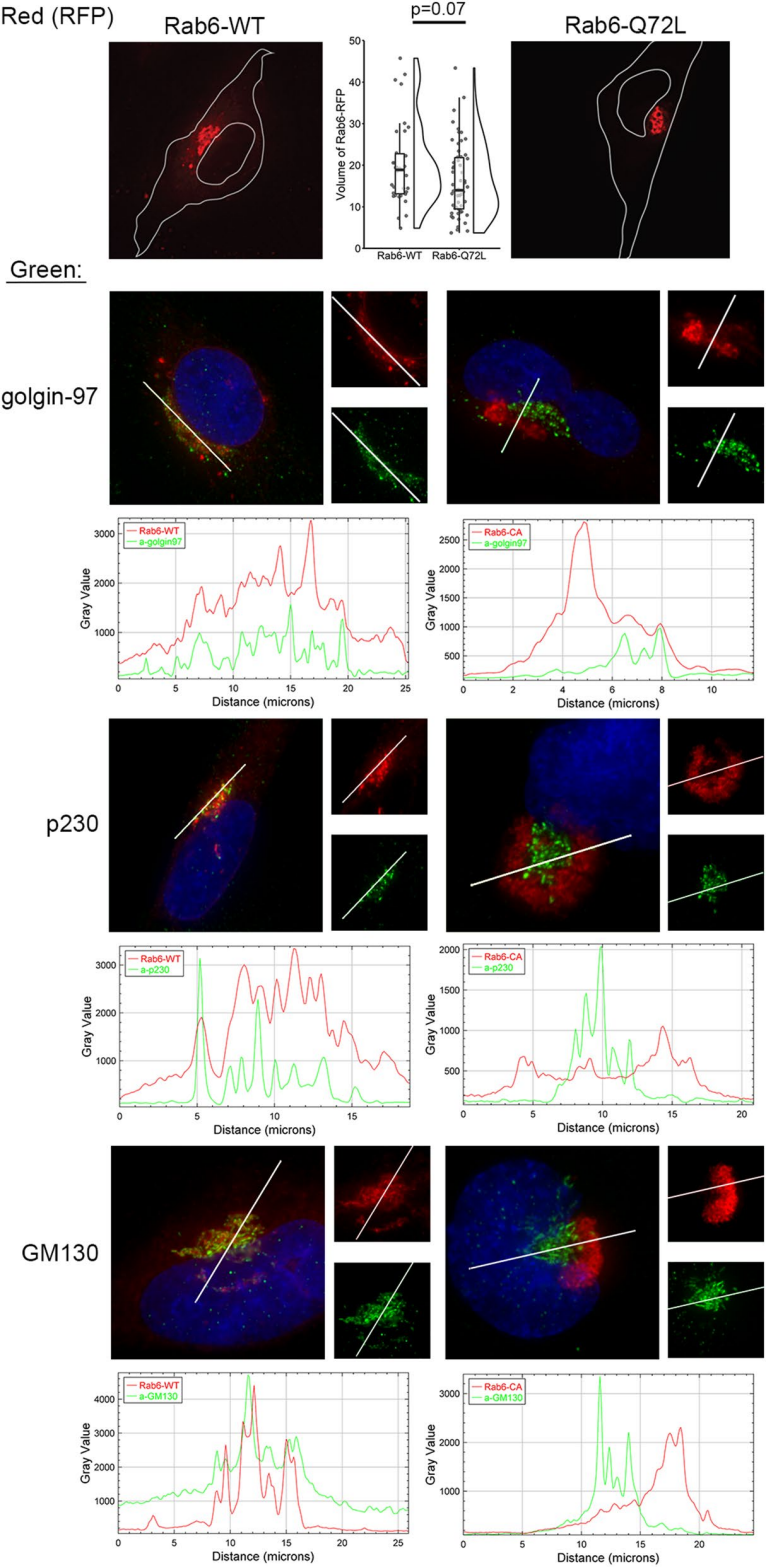
Rab6-Q72L has altered cellular localization relative to the Golgi markers golgin-97, p230, and GM130. BEAS-2B cells were transfected with constructs to express Rab6-WT or Rab6-Q72L fused to RFP (red). After 24 h, cells were fixed and stained with antibodies against golgin-97, p230, and GM130 (green) and DAPI (blue). Imaris was used to quantify the volume of the Rab6 constructs. For the regions indicated by white lines, ImageJ was used to determine the fluorescence intensity across a linear plane in order to demonstrate the different localization of Rab6-WT and -Q72L relative to each of the golgi markers. Images shown are representative of at least 60 cells imaged from at least three independent experiments.

To further understand how Rab6 mediates the recycling of MR1, we generated wild-type (WT) and a mutant (Q72L) Rab6 protein fused to RFP in BEAS-2B cells. The Rab6-Q72L mutant is GTP-locked and constitutively active^26^. As expected, Rab6-WT localized mainly to the Golgi, in areas where it colocalized with the Golgi markers golgin-97, p230, and GM130 (Fig. 5, left). Rab6-Q72L also localized to an area near the Golgi, but in a more dense and tight structure that was largely distinct from golgin-97, p230, and GM130 (Fig. 5, right).The structural change to the localization of Rab6-WT versus Rab6-Q72L was reflected in a significantly different volume of cellular Rab6 (Fig. 5, top), as well as the need to acquire images at different settings to achieve similar emission values to avoid saturation for deconvolution and analysis purposes (Rab6-WT: 100% transmission, 0.05–0.10 s; Rab6-Q72L: 32% transmission, 0.025–0.15 s).

**Figure 6:**
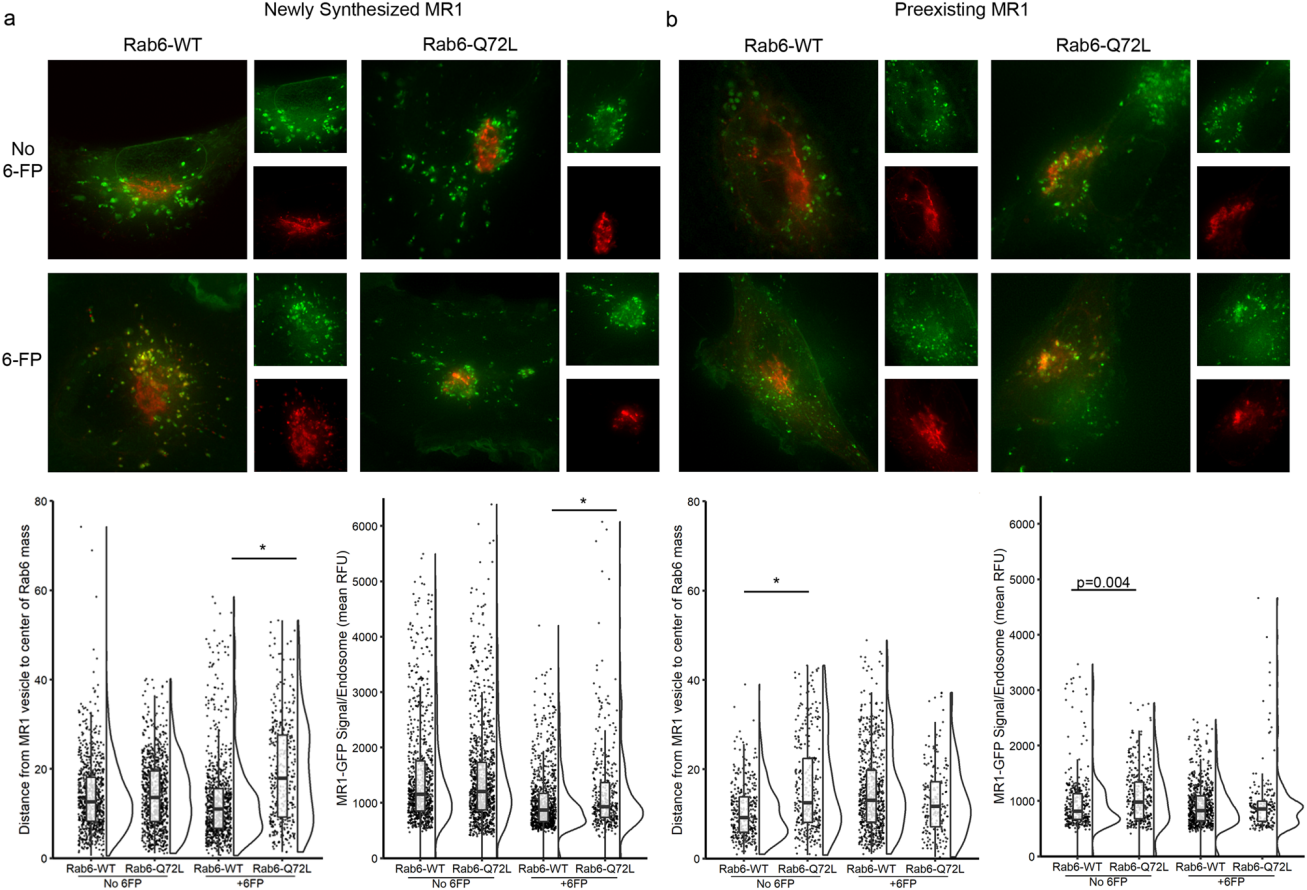
Intracellular localization of MR1 is altered in cells expressing Rab6-Q72L. BEAS-2B:doxyMR1-GFP cells were transfected with constructs for expression of Rab6-WT or Rab6-Q72L fused to RFP, along with doxy and 6-FP. For newly synthesized MR1-GFP, Rab6 constructs were transfected 8 h prior to doxy induction of MR1-GFP expression. For previously synthesized MR1, Rab6 constructs were transfected 24 h after washing doxy from the cells. Cells were imaged live and analyzed using Imaris to quantify the distance (in µm) between each MR1^+^ endosomal compartment and the center of the Rab6-RFP construct mass and the mean relative fluorescence of the MR1 in each compartment. *Indicates p value < 0.001. Results are representative of at least three independent experiments.

These Rab6 constructs were then expressed in the BEAS-2B:doxyMR1-GFP cells expressing newly synthesized or preexisting MR1. When compared with the missense control in Fig. 4A,B, expression of Rab6-WT did not impact the localization of newly synthesized or preexisting MR1 (Fig. 6a,b). In general, the distance between MR1 and the center of both Rab6-RFP masses has a bimodal distribution, which may illustrate MR1 residing in distinct endosomal compartments. In cells treated with 6-FP, more newly synthesized MR1 is located farther from the center of the Rab6-Q72L mass than of the Rab6-WT mass (Fig. 6a, p < 0.001). Although not significant, a similar pattern exists at steady state, where the Rab6-Q72L cells have a larger population of distant MR1 than Rab6-WT cells. Put together, these data suggest that constitutive Rab6 action alters the transport of newly synthesized MR1 residing in post-ER endosomal compartments. Preexisting MR1 is more distant from Rab6-Q72L than Rab6-WT in cells at steady state (p < 0.001); however, there is no significant difference seen in cells treated with exogenous ligand. Together, these data suggest that Rab6 functions in recycling and retrograde transport of MR1 from the cell surface through endosomal pathways to the trans-Golgi network.

## Discussion

MR1 is unique among MHC Class I and Class I-like molecules in that very little MR1 is expressed on the cell surface prior to infection despite ubiquitous expression at the RNA level. This suggests that the antigen processing and presentation pathways for MR1 are distinct from those of other Class I molecules. It is well-established that exogenously added ligands like 6-FP are preferentially loaded on ER-resident MR1 and then translocated to the cell surface, as reviewed in^23^. As with Class I and Class II, however, it is likely that there is more than one pathway by which ligands can be loaded. First, there are distinct pathways by which ligands from intracellular pathogens such as *Mycobacterium tuberculosis* (Mtb) can be loaded on MR1. While Mtb ligands can be loaded on ER-resident MR1, they are also loaded via distinct post-Golgi pathways that involve endosomal trafficking molecules like Rab6 and VAMP4^21^. Additionally, MR1 previously loaded with 6-FP and translocated to the cell surface is recycled to a compartment where it is available for exchange with other exogenously added ligands^25^. Interestingly, ligands from live or fixed *E. coli* are also presented via distinct pathways^24^. Together, these data support the concept that MR1 can use multiple pathways to sample ligands deriving from extracellular and intracellular environments. Here, we sought to better understand the role of Rab6 in defining the MR1 antigen presentation pathway for *Mycobacterium* ligands, using inducible MR1 expression and 6-FP as a surrogate ligand.

We previously demonstrated that silencing of Rab6 resulted in decreased presentation of Mtb ligands by WT BEAS-2B cells to MAIT cells but did not affect translocation of MR1 to the cell surface after addition of 6-FP in cells overexpressing MR1, suggesting that Mtb ligands can be loaded and presented via a different pathway than 6-FP^21^. To assess the role of Rab6 in trafficking of newly synthesized versus preexisting MR1 molecules, we generated a cell line that stably expresses MR1 behind a doxycycline-inducible promoter. By using inducible expression of MR1, we could specifically observe the impact of Rab6 silencing on newly synthesized MR1 molecules versus those already made. While consistent after removal of doxy, the decline in the level of *MR1* transcripts was not as rapid as we had anticipated. The limited time between the disappearance of *MR1* transcript and the loss of detectable MR1-GFP protein suggests that MR1 rapidly transits to the surface, through endosomal recycling pathways, and ultimately to degradation. This relationship between gene and protein expression resulted in challenges in analyzing the effect of Rab6 knockdown on MR1 trafficking. We had to balance the time required to achieve Rab6 silencing with the time it took to increase the probability that the observed MR1 was preexisting and not newly formed, while also maintaining the ability to detect remaining MR1 protein before degradation. Thus, there was a reduced ability to detect significant differences between the Rab6-silenced and control cells in some cases. Nonetheless, because we were able to compare the effects of Rab6 silencing on preexisting MR1 molecules to those that we knew were newly synthesized, resulting in confidence that the data show the biologically relevant impacts of Rab6 silencing.

Our data best support a function for Rab6 in retrograde transport of MR1 from the plasma membrane to the TGN. The phenotype observed in Rab6-silenced cells could be explained by several possible Rab6 functions within this pathway. First, Rab6 could be directly involved in endocytosis from the cell surface, thus when Rab6 is silenced, MR1 would be trapped on the cell surface. Because we do not observe such an increase in surface MR1, our data better support a second possibility where MR1 is endocytosed from the cell surface and then sorted into multiple discrete pathways, some of which are mediated by Rab6. MR1 that is endocytosed from the cell surface could enter pathways where it subsequently recycles back to the cell surface with or without exchange for a new ligand, or it could enter the lysosomal degradation pathway. Our imaging data suggest Rab6 may result in preferential trafficking into one of these pathways. At steady state, expression of constitutively active Rab6-Q72L results in a greater population of preexisting MR1 likely residing in endosomal compartments, but surface translocation (upon treatment with 6-FP) erases this effect. For cells with newly synthesized MR1, this population is only visible after treatment with 6-FP translocates MR1 from the ER to the surface. Together, these findings suggest that Rab6 functions in retrograde trafficking of surface or endosomal MR1. In BEAS-2B cells overexpressing MR1, much of the intracellular MR1 is observed in compartments expressing late endosomal and lysosomal markers such as Rab7 and Lamp1^21^. In contrast, in the C1R lymphoblast cell line overexpressing MR1, a population of MR1 is observed with early endosomal markers like EEA1 in addition to late endosomal markers^22^, so the point in the endosomal pathway where ligands are loaded may be cell type dependent. In our model, Rab6 activity would direct MR1 into a pathway where ligand exchange and recycling to the cell surface can occur. When Rab6 is silenced, MR1 would be directed into compartments where it is unavailable for ligand exchange and recycling to the cell surface. More work will be necessary to determine the phenotype of these different subsets of endosomal compartments observed in our Rab6-silenced cells. This model suggests that microbially-derived MR1 ligands are loaded onto MR1 earlier in endocytosis, prior to trafficking of bacteria to lysosomes for degradation. In the case of Mtb, which inhibits fusion of the phagosome with lysosomes and continues to be metabolically active within the host cell, it might suggest a unique importance for ongoing presentation of Mtb ligands and MR1-dependent MAIT cell responses.

Together, our data and that of our previous work^21,25^ are consistent with a model in which exchange of MR1 ligands, including 6-FP and those derived from bacterial infection, can occur in endosomal trafficking pathways, some of which are Rab6-mediated. This is distinct from what has been seen in C1R lymphoblasts^22^ and suggests the possibility of cell-specific MR1 trafficking and loading pathways for ligands derived exogenously and during intracellular infection. Further exploration of MR1 ligand exchange in epithelial cells and professional antigen presenting cells may reveal the differential physiological role of MR1 presentation in these cell types. The significance of these different pathways in the context of mucosal tissues during bacterial infections remains unknown. Furthermore, perturbation of pathways that impact endosomal recycling of MR1 may also impact the internalization and trafficking of bacteria taken up by the host cell. The impact of silencing trafficking molecules such as Rab6 on the availability of MR1 ligands has yet to be explored. It is likely that the pathways for MR1 antigen presentation are not mutually exclusive. Ligands from different intracellular and exogenous sources may be preferentially, but not exclusively, presented through these pathways. Continued understanding of the mechanisms for presenting MR1 ligands will be critical to understanding how MAIT cell activation is regulated to prevent inappropriate inflammatory responses in mucosal tissues, and also to better target MAIT cells for therapeutics and vaccination.

## Methods

### Human subjects

This study was conducted according to the principles expressed in the Declaration of Helsinki. Study participants, protocols and consent forms were approved by Oregon Health & Science University Institutional Review Board (IRB00000186). Written and informed consent was obtained from all donors. Human participants are not directly involved in the study. Healthy adults were recruited from among employees at Oregon Health & Science University as previously described to obtain human serum^2^.

### Cells and reagents

BEAS-2B bronchial epithelial cells were obtained from the American Type Culture Collection (ATCC CRL-9609) and cultured in DMEM media (Gibco) supplemented with L-glutamine and 10% heat-inactivated fetal bovine serum. The MR1-restricted T cell clone D426G11 was generated and expanded as previously described^2,4^, then cultured in RPMI (Gibco) supplemented with L-glutamine and 10% heat-inactivated human serum. *Mycobacterium smegmatis* Mc^2^155 (ATCC) was cultured in 7H9 broth. Following growth to late log phase, the bacteria were pelleted and the supernatant was passed over a 0.22 micron filter for use as antigen in ELISPOT assays.

The following antibodies were used for fluorescence microscopy and flow cytometry: α-MR1 (26.5, conjugated to PE or APC, Biolegend), α-golgin-97 (CDF4, Invitrogen), α-GM130 (35/GM130, BD Biosciences), α-p230 (15/p230, BD Biosciences), α-mouse IgG, α-rabbit IgG, α-streptavidin AlexaFluors (Life Technologies). Doxycycline (Sigma) was suspended in sterile water and used at 2 μg/mL. 6-formylpterin (6-FP, Schirck’s Laboratories) was suspended in 0.01 M NaOH and used at a final concentration of 100 μM. NucBlue Cell Stain ReadyProbes (ThermoFisher) was used per the manufacturer’s protocol. Restriction enzymes (EcoRI and KpnI) and ligation enzymes were obtained from New England BioLabs.

### Lentivirus production and generation of stable BEAS-2B:doxMR1-GFP cell line

BEAS-2B cells were stably transduced to express MR1 fused to GFP under a doxycycline (dox)-inducible promotor (BEAS-2B:doxMR1-GFP) as previously described^27^. Briefly, low passage HEK 293T cells (ATCC CRS-3216) were co-transfected with the packaging plasmids (psPAXs and pMD2.G) and the pDMLV2.1:doxMR1 construct using Lipofectamine 3000 (Invitrogen). pDMLV2.1:doxMR1 was generated by subcloning the doxMR1-GFP cassette from the pCI:doxMR1 construct used previously for transient transfection^25^ into the pLenti6.2 plasmid. After 48 h, the medium was removed and passed through a 0.45 micron filter to clear cellular debris. The filtrate was then mixed with an equal volume of DMEM containing 8 μg/mL polybrene (Sigma) and used to transduce wild-type (WT) BEAS-2B cells. Cells were cultured for four days in the presence of 2 μg/mL doxy, then sorted using a BD Influx cell sorter on GFP expression. Following cell sorting and culture, the inducible expression of MR1-GFP was validated by flow cytometry and fluorescence microscopy.

### ELISPOT assay

Enzyme-linked immunospot (ELISPOT) assays were performed as previously described^28^. Briefly, WT BEAS-2B or BEAS-2B:doxMR1-GFP cells (5e3 per well) were used as antigen presenting cells and incubated with a titration of *M. smegmatis* supernatant as the antigen in an ELISPOT assay. IFN-γ production by the MAIT cell clone D426G11 (5e3 per well) was used as a readout for MAIT cell activation.

### siRNA silencing

BEAS-2B:doxMR1-GFP cells were plated in 6-well tissue culture plates or 1.5 mm glass-bottom chamber slides at 70% confluency and transfected with 5 nM siRNA (ThermoFisher Scienctific) using HiPerFect Transfection Reagent (Qiagen). Cells were incubated for indicated times prior to use and analysis in assay. A missense siRNA was used as a control in all experiments and gene silencing was validated by RT-PCR.

### Real-time quantitative PCR (RT-PCR)

RNA isolation, cDNA synthesis and RT-PCR were performed as previously described^21,25^ using TaqMan gene expression assays for Rab6 (Hs01042278_m1) and GAPDH (Hs02758991_g1) (LifeTechnologies). All reactions were run in triplicate. Expression data were normalized to GAPDH and calculation of the relative Rab6 expression level was determined using the 2^−ΔΔCT^ method^29^.

### MR1 surface stabilization assay

BEAS-2B:doxMR1-GFP cells were plated in 6- or 12-well tissue culture plates, and treated with siRNA and doxy as indicated. Following a 16 h incubation with 100 μM 6-FP, cells were harvested and stained on ice with α-MR1-PE or -APC (26.5) for 40 min in the presence of 2% human serum, 2% goat serum, and 0.5% FBS. Cells were washed and analyzed live with a Beckman Coulter CytoflexS. All analyses were performed using FlowJo10 (TreeStar).

### Rab6-RFP construct design and transfection

RFP-Rab6 WT and mutant constructs were generated using the pCI ASCI construct as previously described for other genes^21^. Briefly, duplexed DNA oligos encoding RFP in frame with Rab6 or Rab6-Q72L were obtained from LifeTechnologies, ligated into the pCI expression vector using EcoRI and KpnI restriction sites, and the resulting plasmid was expanded in competent *E. coli*. Individual clones were picked and further expanded to validate the plasmid sequence. BEAS-2B cells were transfected with the RFP-Rab6 constructs by nucleofection using the Amaxa Cell Line Nucleofector Kit T (Lonza) and program G-016 on the Lonza Nucleofector 2b. Transfection efficiency was assessed by flow cytometry and fluorescence microscopy.

### Fluorescence microscopy

BEAS-2B cells were seeded in #1.5 glass bottom chamber slides (Nunc) and treated with reagents as indicated. Live cell images were acquired at 37 °C with 5% CO_2_. For antibody staining, cells were washed, fixed with 4% paraformaldehyde, and permeabilized with 0.05% saponin for 30 min prior to antibody staining. Live and fixed cell images were acquired using a high-resolution wide-field CoreDV microscope (Applied Precision) with CoolSNAP ES2 HQ (Nikon). Images were taken in Z stacks in a 1024 × 1024 format using a 60 × Plan Apo N objective (NA 1.42). The researcher was blind to the cell treatment condition. For fixed cell imaging analysis of MR1 localization, cells were selected in an unbiased manner using DAPI staining. For live cell imaging analysis of MR1 localization, cells were selected in an unbiased manner using Rab6 expression. An iterative algorithm was used to deconvolve the images using an optical transfer function of 10 iterations (Softworx, Applied Precision).

### Data analysis

All data were analyzed using Prism 8 (GraphPad). The Shapiro–Wilk test was used to assess normality, and the Mann–Whitney test was used to determine statistical significance. All images were analyzed using Imaris (Bitplane) as in Harriff et al.^21^ except where noted. Briefly, local regions of high fluorescence intensity were algorithmically identified with the “Spots” module and the relative fluorescence intensity was averaged across each region. Rab6-RFP construct regions were similarly analyzed with the “Surfaces” module. Microscope and Imaris settings were kept constant to allow comparison between conditions and the condition of each image was blinded during analysis to prevent bias. Relative fluorescence intensity histograms were created in ImageJ 2.1.0 over linear regions of interest. Raincloud plots were created using R 4.0.0^30,31^ as previously described^32^*.*
